# Nutritional Value and Physicochemical Properties of Male and Female Broad-Breasted Bronze Turkey Muscle

**DOI:** 10.3390/foods13091369

**Published:** 2024-04-29

**Authors:** Anna Czech, Piotr Domaradzki, Mateusz Niedzielak, Joanna Stadnik

**Affiliations:** 1 Department of Biochemistry and Toxicology, Faculty of Animal Sciences and Bioeconomy, University of Life Sciences in Lublin, Akademicka 13, 20-950 Lublin, Poland; anna.czech@up.lublin.pl; 2 Department of Quality Assessment and Processing of Animal Products, Faculty of Animal Sciences and Bioeconomy, University of Life Sciences in Lublin, 20-950 Lublin, Poland; piotr.domaradzki@up.lublin.pl; 3 Dar-Kam Niedzielak, Pietrusy 39, 08-207 Pietrusy, Poland; 4 Department of Animal Food Technology, Faculty of Food Science and Biotechnology, University of Life Sciences in Lublin, Skromna 8, 20-704 Lublin, Poland

**Keywords:** broad-breasted Bronze, meat quality, physicochemical properties, amino acids, fatty acids, oxido-reduction indices

## Abstract

Colored varieties of turkeys, such as the broad-breasted Bronze, not currently subject to intensive breeding work, are kept only in amateur breeding and treated rather as ornamental poultry. They are raised in extensive systems, which undoubtedly affects the quality of the meat obtained. Consumers are looking for meat with specific and unique sensory qualities; hence, the interest in meat from turkeys with a slower-than-typical growth rate, such as the broad-breasted Bronze, is justified. The object of this research was to analyze the physicochemical properties and nutritional value (amino acid, fatty acid, and antioxidant profile) of the breast and thigh muscles of broad-breasted Bronze turkeys with regard to gender. It was shown that gender had little effect on muscle chemical composition, amino acid, and fatty acid content (*p* > 0.05), as well as most oxido-reduction indices. However, significant differences were noted in muscle quality traits such as color brightness (L*; turkeys > indors; *p* = 0.023), proportion of red (a*; turkeys < indors; *p* = 0.048) and yellow (b*; turkeys > indors; *p* = 0.039), and water absorption (turkeys < indors; *p* = 0.009). The type of muscle also had a significant effect on quality characteristics. Higher a*, b*, C*, pH, water absorption, and thermal leakage were shown in the femoral muscle (*p* < 0.001), while L* and h were higher in the pectoral muscle (*p* < 0.001). Turkey meat was characterized by a high proportion of unsaturated fatty acids (MUFA + PUFA ~68%), favorable FA index values, and low lipid oxidation indices. Thigh muscles (especially turkey) were more caloric, and contained significantly (*p* < 0.001) more fat and all major FA groups. Breast muscles (especially of turkeys) were characterized by a high protein content (about 25%) and a high proportion of essential amino acids. The data obtained indicate that broad-breasted Bronze turkeys can provide high-quality meat, and are an excellent option for meeting modern consumer needs.

## 1. Introduction

The dynamic growth of the poultry market is mainly due to the high demand for poultry meat, as well as changes in the eating habits of consumers. Producers are constantly striving to reduce the occurrence of undesirable meat characteristics that have a negative impact on consumer acceptance [1]. The problems of meat quality defects are related to the physiological state of the birds, stress factors, or the rearing technology itself [2]. Currently, the leading housing system for slaughter turkeys is an intensive system, characterized by high stocking rates per unit floor area, which can increase stress and risk a decrease in natural resistance to infection. In the context of these problems, there is increasing discussion of sustainable poultry production. According to scientists, the comfort of the birds and high standards of rearing have a significant impact on obtaining the highest-quality products. Therefore, there is a noticeable return to traditional methods of keeping birds, allowing direct access to fresh air, and green space, and feeding poultry with feed of natural origin [3].

Additionally, the growing awareness of consumers, for whom the housing systems and welfare of poultry are among the most important factors determining purchasing behavior, is an important issue. Consumers are increasingly looking for meat that is not only characterized by nutritional and dietary qualities, but also from so-called “happy animals”, i.e., animals kept in conditions that are close to natural [4]. Literature data indicate that for maintenance in extensive systems, better-adapted slow-growing hybrids should be chosen; as a result, those having better meat quality are becoming a luxury product [5]. Examples include the French brand “Label Rouge”, known worldwide for the quality of its products from the meat of slow-growing birds, as well as the production of birds of local breeds, including turkeys from the US.

There is no “primitive” breed of turkey on the European market, including Poland. The broad-breasted Bronze turkey can be considered such a breed. The population of broad-breasted Bronze turkeys on European markets is small, while in Poland there are about 600 of them, and all of them are on the Dar-Kam Niedzielak Poultry Farm in Pietrusy district of Łosice. The birds are kept on the farm in extensive rearing with access to paddocks, with feeding based on complete feed mixes with the addition of premixes. These turkeys are characterized both by a very favorable proportion of breast muscle in the carcass, and by excellent-quality meat with high marbling.

Moreover, the interest in meat from turkeys with a slower than typical growth rate, such as the Bronze Standard, is justified. It is the growth rate, regardless of the housing system, that influences such technological features as acidity, water-holding capacity, and lightness (L*). Interestingly, the meat from Bronze birds is not darker than that from birds with white feathering [6]. The tenderness of meat may also be influenced by the size of muscle fibers, which are more numerous and larger in diameter in rapidly growing birds than in slowly growing birds [7]. This may also be due to the greater motor skills of birds, which find it easier to move due to their lower body weight. Castellini et al. [8] reported that slower-growing hybrids spend more than twice as much time on the move as standard broilers intended for intensive breeding. Heavier turkeys may also have a higher carcass fat content than lighter birds [9].

Due to the fact that this breed is not very widespread, so far no research has been carried out in terms of meat quality traits and chemical composition. Therefore, it seems justified in this regard to carry out a detailed study to determine the suitability for culinary and processing purposes of the meat of slow-growing broad-breasted Bronze turkeys maintained in extensive systems.

The aim of this study was to evaluate the qualitative properties and nutritional value, including amino acid, lipid, and antioxidant profiles, of breast and thigh muscles of broad-breasted Bronze turkeys with respect to gender.

## 2. Material and Methods

### 2.1. Animals and Preparation

The experiment was performed on 40 broad-breasted Bronze turkeys (20 female and 20 male turkeys) from the Dar-Kam Niedzielak Poultry Farm in Pietrusy district of Łosice. The birds were kept on the farm in extensive rearing with access to paddocks. In addition to access to pasture, they were fed a complete feed mixture in accordance with the nutritional recommendations [10]. Component composition and nutrient content in feed mixtures for turkeys throughout the entire production cycle (week) are presented in Supplementary Materials (Table S1).

After reaching slaughter weight, i.e., about 6.5–7 kg for females and about 10–11 kg for males according to the production cycle, all birds were subjected to the standard slaughter procedure, and the resulting carcasses were refrigerated for 24 h at 4 °C. After cooling, approximately 400–500 g samples were taken from the middle parts of the whole pectoral muscle and thigh, which were transported under refrigeration (4 °C) to the laboratory after being vacuum-packed in PA/PE bags. Determinations of color, pH, thermal leakage, water-holding capacity, and basic chemical composition were performed 48 h post mortem. Muscle samples for determination of fatty acid and amino acid profiles after shredding and vacuum packing were stored at −40 °C for no more than 1 month.

### 2.2. Physicochemical Properties

#### 2.2.1. Chemical Composition

Water, protein, and total fat and ash contents were determined according to AOAC procedures (934.01, 978.04, 930.09, and 930.05 respectively) [11].

#### 2.2.2. Instrumental Color (L*a*b*)

Meat color was evaluated using an X-Rite Color Premiere 8200 spectrophotometer (X-Rite Incorporated, Grand Rapids, MI, USA) according to the recommendations of the American Meat Science Association [12]. A D65 illuminator and a standard 10° observer were used for the determinations. Samples 5 cm thick were used for measurements, and were covered with a single layer of cling film and left for 30 min at 4 ± 1 °C before determination. The instrument before use was standardized against a white ceramic calibration tile with a specification of L* = 95.87, a* = −0.49, and b* = 2.39, which was wrapped in the same cling film used for the meat samples, and a light trap. Color measurements were made in triplicate at randomly selected locations.

#### 2.2.3. pH

The pH was measured in a meat/distilled water (1:10) suspension homogenized for 1 min using a disperser (T25 Basic ULTRA-TURRAX; IKA, Staufen, Germany). The resulting homogenate was left for 15 min at room temperature. The pH was measured using a CPC-501 digital pH meter (Elmetron, Zabrze, Poland) equipped with a composite glass pH electrode (ERH-111; Hydromet, Gliwice, Poland). The pH meter was standardized against buffer solutions at pH 4.00, 7.00, and 9.00 (Avantor Performance Materials, Gliwice, Poland) prior to use.

#### 2.2.4. Water-Holding Capacity (WHC)

Water-holding capacity (WHC) was measured using a centrifuge method. Samples of 50 g of ground meat were homogenized with 50 mL of distilled water for 1 min using a disperser (T25 Basic Ultra-Turrax, IKA, Staufen, Germany). The homogenates were then centrifuged at 1500 rpm for 20 min using an MPW-350R centrifuge (MPW Med-Instruments, Warsaw, Poland). Water-holding capacity was calculated as: WHC = (M1 − M2)/M3 × 100%, where M1—mass of added water (g); M2—mass of supernatant after centrifugation; M3—mass of meat in homogenate (g).

#### 2.2.5. Thermal Drip

Thermal drip was determined by the percentage weight loss of meat after heating samples (20 g ± 2 g) in plastic bags immersed in a water bath (PolyScience, Niles, IL, USA) at 70 °C for 20 min.

#### 2.2.6. The Fatty Acid (FA) Profile

The fatty acid (FA) profile of muscle samples was determined after fat extraction. Methyl esters of FAs (FAMEs) were prepared by transmethylation of fat samples (50 mg) with a mixture of concentrated sulfuric acid (95%) and methanol according to the AOCS Ce 2-66 method [13], using tridecanoic acid (C13:0; 3 mg/mL; 91988, Supelco Inc., Bellefonte, PA, USA) as an internal standard. Separation of FAMEs was performed by gas chromatography according to the description and conditions given by Domaradzki et al. [14]. Identification of individual FAMEs was carried out using commercially available fatty acid methyl ester standards (Supelco Inc., Bellefonte, PA, USA; Larodan AB, Solna, Sweden). The results obtained after prior conversion of FAMEs to fatty acids (FA) are expressed in (1) relative terms, i.e., as the percentage of each fatty acid in the total pool of identified FAs (% of total identified fatty acids), and (2) absolute terms, i.e., in mg/100 g of fresh meat, based on the internal standard used. Among the identified fatty acids, the following groups, ratios, and indices were distinguished: SFAs—saturated fatty acids; OBCFAs—odd and branched-chain fatty acids; MUFAs—monounsaturated fatty acids; PUFAs—polyunsaturated fatty acids; TFAs—trans-configuration fatty acids; the ratio of n-6 to n-3 fatty acids (n-6/n-3); atherogenic index (AI) = [C12:0 + (4 × C14:0) + C16:0]/[n-6 PUFA + n-3 PUFA + MUFA]; thrombogenic index (TI) = [C14:0 + C16:0 + C18: 0]/[(0.5 × MUFA) + (0.5 × n-6 PUFA) + (3 × n-3 PUFA) + n-3/n-6 PUFA]; hypo- to hypercholesterolemic FAs ratio (h/H) = (C18: 1cis + PUFA)/(C12:0 + C14:0 + C16:0); and peroxidation index (PI) = (% monoenoic acid × 0.025) + (% dienic acid × 1) + (% trienoic acid × 2) + (% tetraenoic acid × 4) + (% pentaenoic acid × 6) + (% hexaenoic acid × 8) [15].

#### 2.2.7. The Amino Acids (AA) Content

Amino acid (AA) content was determined using a Sykam Amino Acid Analyzer (Laserchrom HPLC Laboratories Ltd. Inc., Rochester, UK). Before analysis, samples were hydrolyzed with 6 N hydrochloric acid at 110 °C for 24 h.

The Chemical Score (CS) measure of protein quality was defined as the ratio of the content of an exogenous amino acid in the evaluated turkey muscle protein (mg/g protein) to the content of the same amino acid in the reference protein (mg/g protein), and expressed in % [16]. The essential amino acid for which the CS index reaches the lowest (less than 100%) value is referred to as the limiting amino acid, meaning that in the evaluated protein, of all the essential amino acids, it is present in the smallest amount compared to the reference protein.

#### 2.2.8. Oxidation-Reduction Potential (ORP)

In order to assess the total oxidative or reducing capacity of the meat system, the oxidation-reduction potential (ORP) was assessed as described by Nam and Ahn [17]. ORP is one of environmental factors affecting the growth of bacteria and subsequent storage stability of meat. Meat samples (10 g) were homogenized with 30 mL of deionized water for 1 min using a disperser (T25 Basic ULTRA-TURRAX; IKA, Staufen, Germany). ORP measurements were made using a digital pH meter (CPC-501; Elmetron, Zabrze, Poland) set to the millivolt scale equipped with a platinum redox electrode (ERPt-13; Hydromet, Gliwice, Poland).

#### 2.2.9. TBARS Index

The TBARS index was determined using the method of Pikul et al. [18]. The results are expressed in milligrams of MDA (malondialdehyde) in 1 kg of product, according to the following equation: TBARS [mg MDA/kg] = 5.5 absorbance values of the test sample.

#### 2.2.10. Activity of Antioxidant Enzymes

The activity of antioxidant enzymes, i.e., superoxide dismutase (SOD) and catalase (CAT), and the level of superoxide lipid peroxidation products (LOOH), in muscle were determined according to the methods described by Czech et al. [19].

### 2.3. Statistical Analysis

The collected data were analyzed for normal distribution using the Shapiro–Wilk test. Differences between interaction means were verified using one-way analysis of variance with Tukey’s multiple comparisons test. To detail the effect of gender and meat share, data were analyzed using planned contrasts:

TRT—*p*-value for overall effect (group 1–4) (THB vs. TB vs. THTh vs. TTh),
S—*p*-value for gender effect (THB + THTh) vs. (TB + TTh)
P—*p*-value for the effect of muscle type (THB + TB) a (THTh + TTh)

The Statistica 13 package (Dell Software Inc., Round Rock, Texas, TX, USA) was used to statistically process the data. All data are expressed as group means and standard error of the mean (SEM). A significance level of *p* ≤ 0.05 was adopted.

## 3. Results

The number of factors analyzed in the present study was limited and focused mainly on the analysis of the quality and nutritional value of muscle from the broad-breasted Bronze turkey breed, which is rare on the European market. The study took into account factors that are most likely to affect the quality of meat, i.e., the type of muscle (breast vs. thigh) and the sex of the birds [5]. Due to the fact that broad-breasted Bronze turkeys are a slow-growing breed and raised under specific conditions (i.e., free range), it would be difficult to create and make a comparison with a group of intensively reared turkeys; so, in the discussion presented here, the results of other authors’ studies on the technological and chemical quality of poultry meat of different breeds kept in different rearing systems are used for comparison purposes.

### 3.1. Physicochemical Value of Broad-Breasted Bronze Turkey Muscle

In the broad-breasted Bronze turkeys included in the study, gender significantly affected color brightness (L*; TH > T; *p* = 0.023), red (a*; TH < T; *p* = 0.048), and yellow (b*; *p* = 0.039 THB < TB; THTh > TTh), while it had no significant effect on saturation (C*) or hue angle (h) (Table 1). The pH value of the pectoral and thigh muscles in broad-breasted Bronze females and males ranged from 5.86 (TB) to 6.01 (TTh). The pH value was significantly influenced by the type of muscle. Thigh muscles are characterized by a higher pH than pectoral muscles (*p* < 0.001). The thigh muscle of broad-breasted Bronze turkeys (ThTh and TTh) was characterized by higher water-holding capacity, with concomitant higher thermal leakage, compared to the pectoral muscle (*p* < 0.001).

### 3.2. Nutrients in Broad-Breasted Bronze Turkey Muscles

The results obtained for the content of basic nutrients in the muscles of broad-breasted Bronze turkeys are presented in Table 2. The significantly highest protein content was recorded in the pectoral muscle of males (TB; *p* < 0.001), while the fat content was significantly highest in the thigh muscle of males (TTh; *p* < 0.001). The results obtained in the experiment show that the gender of turkeys slaughtered at the same age had no significant effect on the content of essential nutrients (*p* > 0.05). In both female and male turkeys’ pectoral muscle, the protein and amino acid (AA) content was significantly higher compared to that of the thigh muscle (*p* < 0.001), which was reflected in a higher ratio of essential (E) to other AAs (NP; 0.868 vs. 0.763; *p* < 0.001). In all cases, the so-called Chemical Score (CS) measure of protein quality exceeded 100%, ranging (depending on the type of essential amino acid) from 109.3% (Val in TTh) to as high as 690.6% (Trp in TTh).

Of the distinguished groups of fatty acids (FAs) in the muscles of broad-breasted Bronze turkeys, PUFAs accounted for the highest proportion (about 38% on average), followed by MUFAs and SFAs (about 30% each). On average, PUFAs in the muscles of broad-breasted Bronze turkeys accounted for more than half of all unsaturated fatty acids (UFAs), which is beneficial from a dietary point of view. Among them, the highest proportion was recorded for C18:2 n-6 (average 30%), C18:3 n-3 (average 2.4%), and C20:4 n-6 (average 2.6%). Although a significant effect of gender and muscle type on the proportion of individual FAs was shown, the differences were not statistically confirmed (*p* > 0.05) for most of the fatty acid groups distinguished, i.e., SFA, MUFA, TFA, PUFA, or n-6 PUFA. However, there was a significant effect of muscle type and interaction (gender x muscle type) on the proportion of OBCFA and n-3 PUFA, and in the case of n-6 PUFA also an interaction effect. Pectoral muscles (THB and TB) had a significantly higher (*p* < 0.001) proportion of n-3 PUFA acids compared to thigh muscles (THTh and TTh); this was also reflected in the n-6/n-3 ratio, which was significantly lower (*p* < 0.001) in pectoral muscles. As for FA indices, significant differences were noted for the atherogenic index (AI), peroxidation index (PI), and h/H expressing the ratio of hypo- to hypercholesterolemic acids; significantly, the most favorable, i.e., the lowest AI and highest h/H values, were determined in the thigh muscle of females (THTh).

Presenting the fatty acid profile of meat as the percentage (%) of individual FAs in the total pool of all identified FAs (Table 3) is a good method for comparing the results of similar experiments or for the overall assessment of intramuscular fat quality, which illustrates the proportion of nutritionally valuable FAs in an accessible way. However, from a dietary point of view, it is important to provide results in absolute units, i.e., mg/100 g of meat, which will take into account the fat content of muscle tissue, providing precise information about the supply of individual FAs or groups of FAs after consuming a specific portion of meat (Table 4). This is especially important when the meat of the compared research groups differs significantly in the content and/or composition of the lipid fraction. In contrast to the results of the percentage of FAs in the meat of broad-breasted Bronze turkeys (Table 3), their presentation in units of mg/100 g of meat showed that thigh muscles, compared to breast muscles, were characterized by a significantly (on average by about 2.5 times) higher content of almost all (except OBCFA) groups of fatty acids, i.e., SFA, MUFA, TFA, and PUFA, including n-3 and n-6 acids (Table 4). As in the case of percentages, gender had no significant effect on the content of the most important fatty acids and the sum of the distinguished groups of FAs.

### 3.3. Oxidation-Reduction Indices in Muscles of Broad-Breasted Bronze Turkeys

SOD activity was significantly higher (*p* < 0.001) in thigh muscle (THTh and TTh) than in pectoral muscle. There was also a significantly higher concentration of primary lipid oxidation products, i.e., LOOH, in the thigh muscles (*p* < 0.001), and a lower concentration of secondary lipid oxidation products, as assessed by the TBARS index (Table 5), which may indicate that lipid oxidation is significantly slowed in these muscles, despite the significantly higher PUFA content (Table 4).

## 4. Discussion

The chemical composition and physicochemical properties of meat, including pH, water-holding capacity, or thermal leakage, determine both its nutritional value and its suitability for processing. However, these characteristics will be of no importance to consumers if they do not accept meat color, which is one of the key attributes of meat quality [20]. The quality of meat, including its color, is influenced by a number of factors related to both the origin of the birds and the feeding or housing system [1]. The results of studies on meat color differences between fast- and slow-growing hybrids are inconclusive. Although no significant differences were found in research on chicken broilers [21], the present study showed that the muscles of broad-breasted Bronze turkeys were slightly lighter (mean L* = 50), more yellow (mean b* = 8), and less red (mean a* = 4) compared to commercial BIG 6 hybrids (L* approx. 48; b* approx. 3.3; a* approx. 9), and to extensively reared turkeys (local strain turkeys; L* approx. 51; b* approx. 2.5; a* approx. 6.3) [20]. According to Sarica et al. [6], the meat of Bronze turkeys is not darker than that of white-feathered turkeys, which is a desirable trait because modern consumers are far more likely to choose poultry meat characterized by a light color [1].

From the available literature, there are no conclusive results on the effect of gender on meat color. Single studies, such as Gálvez et al. [22], indicate differences only within one trait, i.e., the proportion of red color (a*). The effect of gender on meat color parameters was noted by Sirri et al. [23] and Damaziak et al. [20], according to which the meat of females is lighter (higher L*), less red (lower, a*), and more yellow (higher b*) compared to that of males. According to Sirri et al. [23], this can be attributed to differences in muscle fiber metabolism, which is influenced by sex hormones. According to Eleroğlu et al. [24], the effect of gender may also be related to fat content, due to the greater degree of fat deposition in the meat of females.

Meat quality traits such as tenderness, water-holding capacity, color, juiciness, and shelf life are highly dependent on muscle pH and the associated post-slaughter glycolysis process. The pH value of the pectoral and thigh muscles in broad-breasted Bronze females and males did not differ from the values reported by Damaziak et al. [20] for BIG 6 (intensive rearing) and local strain (extensive rearing) turkeys, and also for hybrid [22] and Bronze/hybrid [6] turkeys. This suggests that genotype or rearing method has no significant effect on the post-slaughter degree of muscle tissue acidification. This is not consistent with the observations of Castellini et al. [3], according to whom the lower pH of meat from organically reared birds is due to better welfare conditions that minimize pre-slaughter stress and thus the degree of glycogen consumption.

However, the pH value is significantly influenced by the type of muscle. Red muscles, i.e., thigh muscles (more susceptible to oxidation), are characterized by a higher pH than white muscles, i.e., pectoral muscles, which are more susceptible to glycolytic processes [25], which was also confirmed in our study (*p* < 0.001).

The thigh muscle of broad-breasted Bronze turkeys (ThTh and TTh) was also characterized by higher water-holding capacity, with concomitant higher thermal leakage, compared to the pectoral muscle (*p* < 0.001). These results are not consistent with the study of Raach-Moujahed et al. [26], according to which higher meat pH values and higher water-holding capacity are accompanied by stronger water binding, and dissolved flavor precursors are more difficult to release when the meat is bitten. However, in the case of thermal leakage, a completely different relationship was already observed, i.e., thigh muscles were characterized by almost twice as much loss as breast muscles.

The genotype and housing system of the birds had a significant effect on the value of thermal leakage and water-holding capacity. The value of these indices was significantly higher compared to those of BIG 6 turkeys, but comparable to those of free-ranging turkeys kept in the extensive system presented by Damaziak et al. [20].

The contents of basic nutrients in the muscles of broad-breasted Bronze turkeys did not differ from those reported by Damaziak et al. [20] and Sarica et al. [6], and ranged from 19.69% (TTh) to 25.31% (TB) for protein and from 1.04% (TB) to 6.42% (TTh) for fat, and was about 1.07% for ash. A significantly higher fat content in the muscles of broad-breasted Bronze turkeys than that obtained in our study was reported by Igenbayev et al. [27], which could be related to the way they were fed during backyard rearing. Differences in the fat content of the meat may also be conditioned by the ambient temperature, as its fluctuations can cause changes in fat deposition [28]. According to Werner et al. [29], fast-growing birds (e.g., BIG 6) have lower protein and higher fat content than slow-growing birds, indicating an effect of genotype on the chemical composition of turkey muscle, although this was not confirmed by the study of Damaziak et al. [20]. The lack of conclusive evidence of an effect of genotype on the basic chemical composition of meat therefore suggests a significant role for other factors such as the feeding or housing system of turkeys [30].

The effect of gender on the chemical composition of poultry meat has been analyzed in detail primarily in chickens. Far less attention has been paid to this issue in turkeys. This is probably due to the much earlier slaughter of female turkeys and, most often, the separate rearing of the two genders. In both female and male turkeys’ pectoral muscle, the protein and amino acid (AA) content was significantly higher compared to that in the thigh muscle, which was reflected in a higher ratio of essential (E) to other AAs. Similar results were obtained by Gálvez et al. [22]. Regardless of the differences shown, both evaluated turkey muscles were characterized by a very high proportion of nutritionally valuable essential amino acids, exceeding their content in the reference protein proposed by FAO/WHO/UNU [16]. As with basic nutrients, gender had no significant effect on amino acid content. This was confirmed by the study by Gálvez et al. [22].

In the muscles of broad-breasted Bronze turkeys, PUFAs accounted for the highest proportion (about 38% on average), followed by MUFAs and SFAs (about 30% each), which was also confirmed in earlier study by Geldenhuys et al. [31] conducted on turkey, goose, and broiler meat. Studies by Batkowska et al. [32] and Drażbo et al. [33] unequivocally show that, regardless of genotype, meat from birds kept in an extensive system has a higher proportion of PUFAs than that from conventionally kept birds (hybrid turkeys, BIG 6, and BUT 9).

n-3 LC-PUFAs are of particular interest because of the limited conversion of 18:3n-3 to 22:6n-3 in humans. In landlocked countries where consumption of marine products is limited, meat from cattle, sheep, pigs, and especially poultry, as a source of these essential acids, can directly contribute to food and nutrition security [34].

The higher level of n-3 FAs in pectoral muscles was consistent with the results obtained by Castellini et al. [3] for free-range birds. Although the n-6/n-3 values obtained in our study were higher than the dietary recommendations (< 4), this is usually dictated by the high proportion of C18:2n-6 in poultry meat [35]. However, it should be noted that the results were comparable to those reported by Batkowska et al. [32], lower than those obtained by Janječić et al. [36] (n-6/n-3 value > 13), but definitely higher than those obtained by Drażbo et al. [33] (n-6/n-3 value ranging from 5 to 9).

Batkowska et al. [32] indicated a more favorable value of health-promoting FA indices in the meat of BUT 9 and BIG 6 extensively reared turkeys, although the results were worse than those obtained in our study, i.e., AI ranging from 0.48 to 0.63; TI, from 0.92 to 1.23; and PI, from 43.67 to 47.70. Similarly, in the study of Goluch et al. [37], less favorable values (except for the TI index) of FA indices were obtained in meat of BIG 6 males fed with feed rich in antioxidants and probiotics than in our study, i.e., for AI from 0.55 to 0.57; TI from 0.63 to 0.67; h/H to 1.26 to 1.41; and PI from 38.4 to 42.1, respectively.

The presence of polyunsaturated PUFA fatty acids in meat can negatively affect oxidative stability [38]. The oxidation-reduction indices indicate that oxidation-reduction potential and TBARS were the highest in females breast muscle (THB) indicating the predominance of oxidative processes in this group. Interestingly, THB significantly showed the lowest content of MUFA and PUFA, including n-3 FA, and the lowest peroxidation index (PI); this indicates the susceptibility of the tissue to oxidation based on its fatty acid composition was significantly lower (more favorable) compared to TB and was not statistically different from that of the other groups. However, the intensity of lipid oxidation does not depend solely on the degree of unsaturation of fatty acids but also on the content of pro- and antioxidants. Regardless of the differences shown, the TBARS values obtained for the muscles of broad-breasted Bronze turkeys were at a significantly lower level than those reported for meat obtained from commercial hybrids fed antioxidant-rich feeds [38].

## 5. Conclusions

The study was conducted to evaluate the quality of meat from extensively reared broad-breasted Bronze turkeys. The study showed that gender had very little effect on muscle chemistry, amino acid, and fatty acid content, as well as most of the oxidation-reduction indices assessed. However, significant differences were noted in muscle quality traits such as color brightness (females > males), proportion of red (females > males) and yellow (females > males) color, and water-holding capacity (females > males).

The type of muscle also had a significant effect on quality characteristics. Higher a*, b*, C*, pH, water-holding capacity, and thermal leakage were shown in the thigh muscle, while L* and h were higher in the pectoral muscle. In general, in terms of dietary value, turkey meat was characterized by a high proportion of unsaturated fatty acids (MUFA + PUFA) and favorable values of health-promoting FA indices, and also showed relatively low lipid oxidation indices. Thigh muscles (especially those of males) were more caloric, and contained significantly more fat and all major FA groups, including SFA, MUFA, PUFA, n-3, and n-6. On the other hand, breast muscles (especially of males) were characterized by high protein content (about 25%), with high nutritional value due to the high proportion of nutritionally valuable essential amino acids.

As a general conclusion, this research provided nutritional and meat quality information regarding broad-breasted Bronze turkey meat.

## Figures and Tables

**Table 1:** Muscle quality traits of broad-breasted Bronze turkey.

Item ^1^	THB	TB	THTh	TTh	SEM ^2^	TRT ^3^	S ^4^	M ^5^
L*	54.42 ^a^	53.82 ^a^	49.64 ^b^	43.42 ^c^	0.760	<0.001	0.023	<0.001
a*	−1.98 ^d^	−0.986 ^c^	5.05 ^b^	7.83 ^a^	0.665	<0.001	0.048	<0.001
b*	4.23 ^d^	5.35 ^c^	10.45 ^a^	7.95 ^b^	0.401	<0.001	0.039	<0.001
C*	4.68 ^b^	5.48 ^b^	11.62 ^a^	11.16 ^a^	0.526	<0.001	0.872	<0.001
h°	115.2 ^a^	101.4 ^b^	64.29 ^c^	45.55 ^d^	4.51	<0.001	0.071	<0.001
pH	5.88 ^b^	5.86 ^b^	5.93 ^ab^	6.01 ^a^	0.014	<0.001	0.327	<0.001
Thermal drip	9.59 ^b^	6.03 ^c^	15.24 ^a^	13.70 ^a^	0.721	<0.001	0.077	<0.001
WHC	33.51 ^b^	44.05 ^a^	46.97 ^a^	46.53 ^a^	0.995	<0.001	0.009	<0.001

^1^ THTh—turkey hen thigh; THB—turkey hen breast; TTh—turkey thigh; TB—turkey breast; T—turkey; TH—turkey hen; Th—thigh; B—breast; ^2^ SEM—standard error of the mean; ^3^ TRT—*p* value for overall effect (group 1–4) (THB vs. TB vs. THTh vs. TTh); ^4^ S—*p* value for sex effect (THB + THTh) vs. (TB + TTh); ^5^ M—difference between type of muscle (THB + TB) vs. (THTh + TTh). ^a–d^ Values in rows with different letters are significantly different at *p* ≤ 0.05.

**Table 2:** Content of basic chemical constituents (g/100 g), energy value (kcal/100 g), and amino acids (AA; mg/g) and nutritional value of protein (Chemical Score, CS) of broad-breasted Bronze turkey muscles.

Item ^1^	THB	TB	THTh	TTh	SEM ^2^	TRT ^3^	S ^4^	M ^5^
Moisture	72.86	73.46	74.55	73.58	0.235	0.072	0.700	0.051
Protein	24.33 ^b^	25.31 ^a^	20.24 ^c^	19.69 ^c^	0.525	<0.001	0.846	<0.001
Fat	2.08 ^c^	1.04 ^c^	4.01 ^b^	6.42 ^a^	0.523	<0.001	0.524	<0.001
Ash	1.05	1.07	1.06	1.10	0.040	0.975	0.721	0.813
Energy value	116.0 ^b^	110.6 ^b^	117.1 ^b^	136.5 ^a^	1.32	0.045	0.133	0.013
His	12.04 ^b^	13.26 ^a^	7.96 ^c^	6.21 ^d^	0.606	<0.001	0.831	<0.001
Ile	9.89 ^a^	10.44 ^a^	9.19 ^b^	7.94 ^c^	0.196	<0.001	0.382	<0.001
Leu	18.05 ^a^	18.65 ^a^	16.70 ^b^	14.91 ^c^	0.301	<0.001	0.330	<0.001
Lys	20.08 ^a^	20.83 ^a^	18.61 ^b^	16.35 ^c^	0.358	<0.001	0.302	<0.001
Met	6.43 ^a^	6.57 ^a^	5.71 ^b^	5.07 ^c^	0.128	<0.001	0.338	<0.001
Cys	2.53 ^a^	2.55 ^a^	2.33 ^b^	2.35 ^b^	0.023	<0.001	0.726	<0.001
Phe	9.31 ^ab^	9.61 ^a^	8.68 ^b^	7.90 ^c^	0.138	<0.001	0.391	<0.001
Thr	13.03 ^a^	13.21 ^a^	12.10 ^b^	10.77 ^b^	0.202	<0.001	0.158	<0.001
Val	10.75 ^b^	11.29 ^a^	9.63 ^c^	8.74 ^d^	0.213	<0.001	0.689	<0.001
Trp	8.29	8.86	8.81	8.49	0.231	0.222	0.561	0.213
**∑ Essential AA**	**110.4 ^b^**	**115.3 ^a^**	**94.72 ^c^**	**88.71 ^d^**	**2.28**	**<0.001**	**0.903**	**<0.001**
Asp	23.32 ^a^	24.31 ^a^	21.31 ^b^	19.11 ^b^	0.417	<0.001	0.478	<0.001
Ser	8.60 ^a^	8.77 ^a^	8.05 ^b^	7.52 ^b^	0.104	<0.001	0.400	<0.001
Glu	39.02 ^a^	39.58 ^a^	37.40 ^b^	34.47 ^b^	0.415	<0.001	0.158	<0.001
Pro	11.39 ^a^	11.65 ^a^	14.38 ^b^	15.07 ^b^	0.342	<0.001	0.503	<0.001
Gly	9.84	8.65	9.32	9.53	0.401	0.777	0.550	0.826
Ala	13.23	12.16	12.29	11.10	0.370	0.253	0.130	0.183
Tyr	10.08 ^a^	10.47 ^a^	8.39 ^b^	7.10 ^c^	0.284	<0.001	0.441	<0.001
Arg	14.28 ^a^	14.94 ^a^	13.17 ^b^	12.28 ^c^	0.214	<0.001	0.794	<0.001
**∑ Non-essential AA**	**129.8 ^a^**	**130.5 ^a^**	**124.3 ^b^**	**116.2 ^c^**	**1.30**	**<0.001**	**0.162**	**<0.001**
N/P	0.851 ^b^	0.884 ^a^	0.762 ^c^	0.764 ^c^	0.012	<0.001	0.468	<0.001
**∑ AA**	**240.2 ^b^**	**245.8 ^a^**	**219.0 ^c^**	**204.9 ^d^**	**3.47**	**<0.001**	**0.551**	**<0.001**
Chemical Score [%]								
His	334.2 ^b^	360.0 ^a^	242.3 ^c^	201.9 ^d^	16.96	<0.001	0.795	<0.001
Ile	137.3 ^b^	141.6 ^a^	139.9 ^ab^	129.2 ^c^	4.39	<0.001	0.132	0.015
Leu	127.4 ^a^	128.6 ^a^	129.3 ^a^	123.3 ^b^	2.79	<0.001	0.051	0.161
Lys	185.8 ^a^	188.4 ^a^	188.9 ^a^	177.4 ^b^	5.70	<0.001	0.038	0.067
Met	167.3 ^a^	167.2 ^a^	162.9 ^a^	154.6 ^b^	5.99	<0.001	0.077	<0.001
Cys	175.9 ^b^	173.1 ^b^	177.4 ^b^	191.0 ^a^	7.11	<0.001	0.100	0.001
Met + Cys	169.7 ^a^	168.8 ^ab^	166.9 ^ab^	164.5 ^b^	5.86	0.023	0.233	0.006
Phe + Tyr	212.5 ^a^	215.0 ^a^	205.0 ^b^	192.6 ^c^	6.97	<0.001	0.194	<0.001
Thr	235.9 ^ab^	233.7 ^bc^	240.2 ^a^	228.5 ^c^	8.53	<0.001	<0.001	0.829
Trp	575.1 ^c^	601.2 ^b^	289.6 ^d^	690.6 ^a^	36.88	<0.001	<0.001	0.120
Val	114.8 ^ab^	117.8 ^a^	112.7 ^ab^	109.3 ^b^	3.12	0.005	0.929	0.003

^1–5^ see footer Table 1; N/P—ratio of essential to non-essential amino acids (AA). ^a–d^ Values in rows with different letters are significantly different at *p* ≤ 0.05.

**Table 3:** Profile (% of total fatty acids), proportions, and fatty acid indices in broad-breasted Bronze turkey muscles.

Item ^1^	THB	TB	THTh	TTh	SEM ^2^	TRT ^3^	S ^4^	M ^5^
C10:0	0.040	0.037	0.034	0.038	0.001	0.223	0.932	0.261
C12:0	0.699 ^a^	0.631 ^b^	0.657 ^ab^	0.686 ^ab^	0.010	0.039	0.312	0.756
C14:0	1.05	0.999	1.04	1.08	0.010	0.096	0.615	0.072
C16:0	20.77	19.90	19.23	19.87	0.096	0.094	0.232	0.120
C18:0	9.92 ^a^	9.10 ^a^	8.98 ^b^	8.23 ^b^	0.195	0.048	0.594	0.018
C20:0	0.069	0.064	0.080	0.067	0.003	0.110	0.075	0.145
C22:0	0.042	0.043	0.046	0.024	0.002	0.051	0.106	0.246
C24:0	0.021	0.010	0.027	0.016	0.002	0.155	0.038	0.234
**∑SFA**	**29.90**	**30.78**	**30.09**	**30.00**	**0.174**	**0.281**	**0.271**	**0.415**
C15:0 anteiso	0.025 ^b^	0.026 ^b^	0.024 ^b^	0.035 ^a^	0.001	<0.001	0.005	0.084
C15:0	0.146	0.145	0.148	0.150	0.001	0.618	0.895	0.203
C17:0 iso	0.405 ^ab^	0.567 ^a^	0.315 ^b^	0.254 ^b^	0.032	<0.001	0.436	<0.001
C17:0 anteiso	0.018	0.006	0.018	0.015	0.001	0.058	0.037	0.263
C17:0	0.186	0.187	0.172	0.203	0.009	0.705	0.373	0.967
C17:1 c-7	0.017	0.007	0.006	0.009	0.001	0.082	0.360	0.179
C17:1 c-9	0.157 ^b^	0.129 ^c^	0.182 ^a^	0.166 ^ab^	0.005	<0.001	0.018	<0.001
**∑OBCFA**	**0.954 ^ab^**	**1.07 ^a^**	**0.865 ^b^**	**0.830 ^b^**	**0.027**	**0.002**	**0.470**	**0.001**
C14:1 c-9	0.125 ^a^	0.100 ^ab^	0.096 ^b^	0.127 ^a^	0.005	0.016	0.744	0.864
C16:1 c-7	0.388	0.348	0.368	0.374	0.005	0.054	0.115	0.797
C16:1 c-9	2.82	2.16	2.09	2.58	0.109	0.043	0.708	0.495
C16:1 c-13	0.027	0.011	0.017	0.017	0.001	0.120	0.103	0.756
C16:1 c-15	0.184 ^b^	0.317 ^a^	0.117 ^b^	0.103 ^b^	0.020	<0.001	0.147	<0.001
C16:1 c-other	0.025 ^b^	0.042 ^a^	0.020 ^b^	0.003 ^c^	0.003	<0.001	0.980	<0.001
C18:1 c-9	24.63 ^b^	24.04 ^b^	25.49 ^a^	25.76 ^a^	0.048	0.092	0.165	0.036
C18:1 c-10	0.045	0.046	0.059	0.051	0.002	0.073	0.404	0.023
C18:1 c-11	1.85 ^a^	1.79 ^a^	1.60 ^b^	1.59 ^b^	0.028	<0.001	0.498	<0.001
C18:1 c-13	0.039	0.035	0.042	0.028	0.002	0.252	0.072	0.692
C20:1 c-9	0.027 ^a^	0.003 ^b^	0.027 ^a^	0.020 ^a^	0.002	0.017	0.020	0.092
C20:1 c-11	0.222 ^ab^	0.204 ^b^	0.242 ^a^	0.235 ^a^	0.004	0.004	0.151	0.002
C22:1 c-11	0.099 ^b^	0.176 ^a^	0.095 ^b^	0.104 ^b^	0.009	0.001	0.015	0.037
C24:1 c-15	0.032 ^ab^	0.058 ^a^	0.044 ^ab^	0.022 ^b^	0.004	0.016	0.779	0.188
**∑MUFA**	**31.93**	**29.32**	**30.31**	**31.01**	**0.401**	**0.122**	**0.244**	**0.970**
C15:1 t-9	0.014	0.010	0.027	0.024	0.002	0.043	0.444	0.005
C16:1 t-9	0.039 ^a^	0.000 ^b^	0.030 ^a^	0.011 ^b^	0.004	<0.001	<0.001	0.579
C18:1 t-6/7	0.059 ^b^	0.091 ^a^	0.057 ^b^	0.070 ^b^	0.004	<0.001	0.001	0.118
C18:1 t-9	0.059	0.056	0.056	0.049	0.002	0.473	0.285	0.267
C18:1 t-11	0.039	0.045	0.038	0.034	0.001	0.355	0.780	0.166
C18:2 trans	0.043	0.030	0.035	0.031	0.002	0.600	0.252	0.651
**∑TFA**	**0.237**	**0.232**	**0.243**	**0.219**	**0.009**	**0.820**	**0.421**	**0.860**
C18:2 n-6	29.41 ^b^	28.70 ^b^	31.62 ^a^	31.28 ^a^	0.300	<0.001	0.398	<0.001
C18:3 n-6	0.079	0.092	0.088	0.094	0.003	0.403	0.152	0.417
C20:2 n-6	0.266 ^b^	0.307 ^a^	0.256 ^b^	0.266 ^b^	0.006	0.003	0.025	0.026
C20:3 n-6	0.142 ^b^	0.200 ^a^	0.132 ^b^	0.138 ^b^	0.008	0.002	0.036	0.019
C20:4 n-6	1.69 ^b^	3.65 ^a^	2.85 ^b^	2.46 ^b^	0.238	0.005	0.024	0.048
C22:4 n-6	0.575 ^b^	1.02 ^a^	0.434 ^b^	0.448 ^b^	0.057	<0.001	0.043	0.001
C22:5 n-6	0.161 ^b^	0.325 ^a^	0.134 ^b^	0.129 ^b^	0.020	<0.001	0.045	0.003
**∑n-6**	**33.62 ^b^**	**35.14 ^ab^**	**35.51 ^a^**	**34.82 ^ab^**	**0.247**	**0.029**	**0.415**	**0.115**
C18:3 n-3	2.44 ^a^	2.17 ^b^	2.38 ^ab^	2.58 ^a^	0.041	0.001	0.700	0.035
C20:3 n-3	0.022	0.012	0.024	0.018	0.001	0.289	0.087	0.405
C20:5 n-3	0.054	0.082	0.055	0.048	0.005	0.064	0.304	0.104
C22:5 n-3	0.472 ^ab^	0.658 ^a^	0.313 ^b^	0.278 ^b^	0.039	<0.001	0.350	<0.001
C22:6 n-3	0.361 ^b^	0.538 ^a^	0.211 ^c^	0.204 ^c^	0.033	<0.001	0.211	<0.001
**∑n-3**	**3.35 ^ab^**	**3.46 ^a^**	**2.98 ^c^**	**3.13 ^bc^**	**0.051**	**<0.001**	**0.211**	**<0.001**
**∑PUFA**	**36.97**	**38.60**	**38.49**	**37.94**	**0.264**	**0.104**	**0.314**	**0.429**
n-6/n-3	10.05 ^c^	10.16 ^c^	11.96 ^a^	11.15 ^b^	0.177	<0.001	0.338	0.000
AI	0.362 ^a^	0.361 ^a^	0.350 ^b^	0.361 ^a^	0.002	0.034	0.204	0.072
TI	0.677	0.702	0.698	0.688	0.005	0.308	0.434	0.736
h/H	2.98 ^b^	3.00 ^b^	3.14 ^a^	3.02 ^b^	0.018	0.002	0.187	0.007
PI	57.13 ^b^	67.45 ^a^	55.70 ^b^	53.97 ^b^	1.53	0.002	0.166	0.011

^1−5^ see footer Table 1. ^a–d^ Values in rows with different letters are significantly different at *p* ≤ 0.05.

**Table 4:** Fatty acid content (mg/100 g of meat) in the muscles of broad-breasted Bronze turkeys *.

Item ^1^	THB	TB	THTh	TTh	SEM ^2^	TRT ^3^	S ^4^	M ^5^
C16:0	556.2 ^b^	349.3 ^b^	1034.6 ^a^	1235.0 ^a^	92.95	<0.001	0.987	<0.001
C18:0	213.5 ^b^	131.7 ^b^	467.9 ^a^	494.3 ^a^	38.67	<0.001	0.729	<0.001
**∑SFA**	**823.5 ^b^**	**513.3 ^b^**	**1604.3 ^a^**	**1849.0 ^a^**	**140.0**	**<0.001**	**0.910**	**<0.001**
C17:0 iso	10.74 ^b^	9.12 ^b^	16.05 ^a^	14.40 ^a^	0.682	<0.001	0.239	<0.001
**∑OBCFA**	**26.08**	**39.73**	**45.80**	**50.53**	**5.65**	**0.475**	**0.428**	**0.182**
C16:1 c-9	80.80 ^bc^	39.40 ^c^	116.2 ^ab^	165.4 ^a^	12.89	0.001	0.884	0.001
C18:1 c-9	719.7 ^bc^	424.3 ^c^	1368.1 ^ab^	1599.1 ^a^	125.4	<0.001	0.901	<0.001
**∑MUFA**	**884.9 ^bc^**	**517.6 ^c^**	**1628.9 ^b^**	**1929.8 ^a^**	**149.8**	**<0.001**	**0.915**	**<0.001**
**∑TFA**	**6.62 ^bc^**	**3.90 ^c^**	**12.58 ^ab^**	**14.75 ^a^**	**1.24**	**0.001**	**0.916**	**<0.001**
C18:2 n-6	795.9 ^b^	498.2 ^b^	1649.0 ^a^	1904.2 ^a^	148.2	<0.001	0.945	<0.001
C20:4 n-6	80.64 ^b^	72.17 ^b^	138.5 ^a^	138.4 ^a^	7.01	<0.001	0.766	<0.001
**∑n-6**	**910.0 ^b^**	**602.1 ^b^**	**1839.7 ^a^**	**2105.7 ^a^**	**157.6**	**<0.001**	**0.949**	**<0.001**
C18:3 n-3	67.41 ^bc^	38.73 ^c^	125.9 ^ab^	159.4 ^a^	12.24	<0.001	0.924	<0.001
C20:5 n-3	1.51 ^b^	1.33 ^b^	2.70 ^a^	2.82 ^a^	0.183	<0.001	0.930	<0.001
C22:5 n-3	12.74 ^bc^	10.60 ^c^	15.25 ^a^	14.78 ^ab^	0.481	<0.001	0.179	<0.001
C22:6 n-3	9.75 ^ab^	8.65 ^b^	10.27 ^a^	11.17 ^a^	0.268	0.003	0.859	0.002
**∑n-3**	**92.04 ^bc^**	**59.57 ^c^**	**155.3 ^ab^**	**189.5 ^a^**	**13.05**	**<0.001**	**0.974**	**<0.001**
**∑PUFA**	**1002.0 ^b^**	**661.7 ^b^**	**1995.0 ^a^**	**2295.2 ^a^**	**170.5**	**<0.001**	**0.955**	**<0.001**

* the table shows the most important FA with the highest content in a given group; ^1–5^ see footer Table 1. ^a–d^ Values in rows with different letters are significantly different at *p* ≤ 0.05.

**Table 5:** Oxidation-reduction indices of broad-breasted Bronze turkey muscles.

Item ^1^	THB	TB	THTh	TTh	SEM ^2^	TRT ^3^	S ^4^	M ^5^
ORP	199.6 ^a^	172.4 ^ab^	152.9 ^b^	140.6 ^b^	5.62	<0.001	0.515	<0.001
CAT U/g	307.6 ^b^	401.2 ^a^	287.9 ^b^	350.5 ^ab^	12.69	0.002	0.001	0.170
SOD U/g	246.4 ^b^	258.3 ^b^	1184.9 ^a^	1261.9 ^a^	102.5	<0.001	0.834	<0.001
TBARS	1.70 ^a^	0.041 ^c^	0.083 ^b^	0.094 ^b^	0.231	<0.001	0.044	0.091
LOOH [μM/g]	21.77 ^b^	19.37 ^b^	29.43 ^a^	27.89 ^a^	0.923	<0.001	0.296	<0.001

^1–5^ see footer Table 1. ^a–d^ Values in rows with different letters are significantly different at *p* ≤ 0.05.

## Data Availability

The data presented in this study are available on request from the corresponding author. The data are not publicly available due to privacy restrictions.

## Supplementary Materials

The following supporting information can be downloaded at: https://www.mdpi.com/article/10.3390/foods13091369/s1, Table S1: Component composition (g) and nutrient content (g/kg) in feed mixtures for turkeys throughout the entire production cycle (week).
