# Supplementary material for: Nutritional Value and Physicochemical Properties of Male and Female Broad-Breasted Bronze Turkey Muscle
Source: Foods. 2024 Apr 29;13(9):1369. doi: 10.3390/foods13091369 (PMC11082980; doi:10.3390/foods13091369)
Supplement: Supplementary file 1 [file foods-13-01369-s001.zip › foods-2924692-supplementary.pdf]

Table S1. Component composition (g) and nutrient content (g/kg) in feed mixtures for turkeys throughout the entire production cycle (week)

**Component**	**1-3**	**4-9**	**10-13**	**14-15**	**16-19**	**20-25**
Wheat	493	584.35	567.42	608.04	595.32	669.65
Barley	30	30	30	30	30.00	30
Soybean meal	355.8	249	133	67	0	0
Wheat bran	50	0	114	112	220	220
Sunflower meal	0	50	60	80	120	54
Rapeseed meal	0	32	60	70	9	0
Soybean oil	10	10	8	8	0	0
Fishmeal	18	0	0	0	0	0
Monocalcium phosphate	10.8	6.3		0	0	0
Fodder chalk	9.4	10.8	8.1	7.8	8.5	8.4
Dicalcium phosphate	6	8	7	4	2.5	3.9
Avum hemoglobin	0	5	0	0	0	0
L-Lysine SO4	0	0	0	0	3.7	0
Mineral and vitamin premix	17	14.55	12.48	13.16	10.98	14.05
TOTAL (kg)	**1 000**	**1 000**	**1 000**	**1 000**	**1000**	**1 000**
Chemical composition						
Crude ash	70.44	62.18	52.96	47.68	46.89	44.55
Total protein	237.9	220.0	189.9	174.9	154.9	136.0
Total fat	30.84	29.14	30.33	30.74	24.94	25.23
Crude fiber	32.2	37.68	47.64	50.22	55.41	45.55
Lysine	12.8	11.95	9.22	8.28	7.16	6.06
Methionine	4.26	4.39	3.81	3.69	3.41	2.95
Threonine	9.45	7.90	6.65	6.00	4.91	4.13
Tryptophan	3.14	2.86	2.45	2.21	1.90	1.68
Calcium	12.02	9.49	7.09	6.19	5.60	5.60
Total phosphorus	8.89	7.09	6.18	5.62	5.69	5.33
Available phosphorus	5.10	3.70	2.50	2.10	1.90	1.91
Sodium	1.24	1.27	1.31	1.30	1.35	1.33
